# Associations between Facial Emotion Recognition and Mental Health in Early Adolescence

**DOI:** 10.3390/ijerph17010330

**Published:** 2020-01-03

**Authors:** Gabrielle Simcock, Larisa T. McLoughlin, Tamara De Regt, Kathryn M. Broadhouse, Denise Beaudequin, Jim Lagopoulos, Daniel F. Hermens

**Affiliations:** 1 Sunshine Coast Mind and Neuroscience Thompson Institute, University of the Sunshine Coast, Birtinya 4575, Australia; lmclough@usc.edu.au (L.T.M.); kbroadhouse@usc.edu.au (K.M.B.); dbeaudeq@USC.EDU.AU (D.B.); Jim.Lagopoulos@usc.edu.au (J.L.); dhermens@usc.edu.au (D.F.H.); 2 School of Psychology, University of the Sunshine Coast, Sippy Downs 4556, Australia; tderegt@usc.edu.au

**Keywords:** facial emotion recognition, adolescence, mental health, emotion processing bias

## Abstract

Research shows that adolescents with mental illnesses have a bias for processing negative facial emotions, and this may play a role in impaired social functioning that often co-exists with a mental health diagnosis. This study examined associations between psychological and somatic problems and facial emotion recognition in early adolescence; as any processing biases in this age-group may be an early indicator of later mental illnesses. A community sample of 40 12-year-olds self-rated their symptoms of anxiety, depression, and somatization via two mental health screeners. They also completed a computerized emotion recognition task in which they identified photographs of 40 faces showing expressions of anger, fear, sadness, happiness, or neutral expression. Results showed that increased symptoms of anxiety, depression, and somatization were significantly associated with fewer correct responses to angry expressions. These symptoms were also associated with faster and more accurate recognition of fearful expressions. However, there was no association between mental health and recognition of sad affect. Finally, increased psychological and/or somatic symptomology was also associated with better identification of neutral expressions. In conclusion, youth with increased psychological and/or somatic problems exhibited a processing bias for negative anger and fear expressions, but not sadness. They showed better processing of neutral faces than youth with fewer psychological and/or somatic problems. Findings are discussed in relation to indicators of mental illnesses in early adolescence and the potential underpinning neural mechanisms associated with mental health and emotional facial recognition.

## 1. Introduction

Face perception is of critical importance in social interactions. In addition to providing information regarding age, gender, race, and identity, facial expressions provide important cues about thoughts and emotions [1]. Correct processing and interpretation of emotions conveyed by facial expressions is crucial as it ensures successful interpersonal communication. Decades of facial recognition research has shown that discriminating facial expressions is an innate ability evident in infancy [2], and it is refined across childhood [3,4] and adolescence [1]. There are six universal facial expressions which correspond to key individual emotions: happy, sad, anxious, disgust, fear, and surprise [5]. Furthermore, and specific to the purpose of the current paper, research demonstrates that anomalies in facial recognition ability are evident in individuals with mental health problems [6].

Emotional dysfunction, including impaired emotion recognition, is a characteristic psychopathology, with individuals showing deficits in the ability to appropriately read signals transmitted by others’ expressions [7,8]. It is thought that this deficit accounts, in part, for the poor social and interpersonal functioning observed in youth [9] and adults experiencing a variety of mental illness, including anxiety and mood disorders [6]. A growing body of research indicates that individuals with mental illness exhibit a processing bias for negative facial expressions [6], and that this bias may be apparent even prior to the onset of a mental disorder. Some studies show that individuals with mental illness experience hypervigilant recognition of negative affect expressions [10]; whereas other studies report less accurate or slower processing [6,11]. In contrast, some research fails to detect differences in facial recognition between those with mental illnesses and healthy controls (HC) [9]. Where differences are detected, the literature suggests that facial emotion-processing impairments may differ as a function of the type of mental illness and specific negative facial expression.

McClure, Pope [9] and colleagues reported that youth with bipolar disorder, but not HC or youth with anxiety, were prone to misidentify photos of children’s (but not adults’) facial expressions of happiness, sadness, or fear as angry. Similarly, a study of youth with comorbid bipolar and anxiety disorders reported a bias towards negative affect faces with no such bias shown in bipolar youth without an anxiety disorder or the HC [12]. Waters, Henry [13] and colleagues found that children with severe generalized anxiety disorder had an attentional bias for angry faces compared to children with mild anxiety and HC. In contrast, one study found poorer recognition of fear expressions by youth with major depressive disorder and poorer recognition of anger in dysthymic youth compared to HC, with no between-group differences for non-negative facial expressions [14]. Similarly, adolescents with anxiety disorders made more errors recognizing negative facial expressions (anger, fear) and had more accurate recognition of neutral faces compared to HC [15].

One study also demonstrated a processing bias for sad facial expressions in depressed youths, who mistook other expressions (e.g., happy, angry, fearful) for sadness; whereas HC mistook these other expressions for happiness [16]. Similar facial recognition impairments are evident in youth with psychopathic tendencies, who showed difficulty accurately recognizing sad and fearful expressions [17].

In contrast, maltreated children with and without PTSD symptoms showed faster identification of all facial expressions, especially fear expressions, compared to non-maltreated children [10]; and boys at familiar risk for depression showed enhanced processing of sad expressions [18]. Whilst it is unclear whether negative processing bias of facial expressions exists prior to a diagnosis of an affective disorder, the identification of such phenotypes for an emerging youth mental illness is important in early diagnosis and treatment.

Adolescence is a sensitive period for the onset of mental health problems, with over half of adult mental illnesses emerging by 14 years of age [19]. Australian statistics show that 1 in 7 young people have an anxiety disorders and 1 in 16 in have depression, with pre-clinical psychological distress symptoms often emerging prior to diagnosis in early adolescence [20]. These youth mental health statistics are consistent with those in other Western countries [21]. As measures of general mental health used in clinical care are highly correlated with common mental illnesses, it is possible that young people with elevated psychological problems, prior to a clinical diagnosis, may already exhibit atypical recognition of negative faces.

The Longitudinal Adolescent Brain Study (LABS) is a prospective cohort study conducted at the Sunshine Coast Mind and Neuroscience Thompson Institute (SCMNTI), University of the Sunshine Coast (USC), Queensland, Australia. This study tracks the mental health, psychosocial, and neurobiological function of adolescents across high school to establish the onset and trajectory of mental health problems during this dynamic phase of development.

The aim of the current study is to examine associations between facial recognition ability and mental health in the LABS cohort in their first year of high school (Grade 7; aged 12 years). We hypothesize that young people with poorer mental health will show a processing bias (fewer correct responses, slower reaction times, more false positive misattributions) for faces with negative or threat affect (angry, fearful, sad) compared to young people with lower levels of psychological distress.

## 2. Materials and Methods 

### 2.1. Participants

Commencing June 2018, adolescents were recruited into LABS via publicity drives and advertising at local community events and by word-of-mouth. Participants were included in the study if they resided in the Sunshine Coast, Queensland, Australia, were in their first year of high school (Grade 7) and were 12-year-old when booking into their first assessment. Participants completed a self-report questionnaire on their psychological wellbeing, a battery of cognitive assessments, a neuropsychiatric screener and neuroimaging scans. Follow-up assessments utilizing the same methodology are scheduled at 4-monthly intervals across five years of high school. Using these methods, to date, 40 adolescents (20 girls and 20 boys) aged 12 years (M = 12.67 years, SD = 0.31) have participated in the initial LABS assessment and are included in the current manuscript. Data on the mental health measures (K10, SPHERE-12) and social cognition (facial emotion recognition) will be reported here. LABS received ethical approval from the University of the Sunshine Coast (HREC #A181064) and all participants and their caregivers provided informed consent prior to participation. Participants were thanked for their time with at $20 gift voucher.

### 2.2. Mental Health

The Kessler Psychological Distress Scale (K10) [22] was used to obtain a global measure of anxious and depressive symptoms during the past four weeks. The K10 is a self-reported tool and is widely used as a screener for general psychological distress in adult and adolescent populations in primary care settings. Participants rated 10 statements on five-point Likert scale (1 = ‘none of the time’ and 5 = ‘all of the time’). Total scores range from 10 to 50 and established cut-offs indicate the likelihood of a mild (scores 21–24), moderate (scores 25–29), or severe (scores 30+) mental disorder. This tool has good psychometric properties in an adolescent population [23].

The Somatic and Psychological Health Report (SPHERE-12; [24]) was used to obtain a measure of psychological and somatic symptoms during the past few weeks. The SPHERE-12 is a self-report tool utilized as a mental health screener in primary care. Participants rated 12 statements on a 0 to 2 scale (0 = ‘never or some of the time’, 1 = ‘a good part of the time’, 2 = ‘most of the time’), with total scores ranging from 0 to 24. Scores of 6–11 indicate some symptomology and scores 12 and above indicate high symptomology. Additionally, two broadly defined sub-scales are reported on as they tease apart psychological versus somatic mental health symptoms. The PSYCH subscale (items 1–6; PSYCH score ≥ 2 and SOMA < 3) assesses symptoms of anxiety and depression for a possible mental disorder and the SOMA subscale (items 7–12; SOMA ≥ 3 and PSYCH < 2) assesses symptoms of fatigue for a possible somatoform disorder [25]. The sub-scales have very high internal consistency (PSYCH 0.9; SOMA 0.8) and test–retest reliability (PSYCH 0.81; SOMA 0.8) [24]. There is also a narrowly defined depression caseness (PSYCH ≥ 2 and SOMA ≥ 3) [25].

### 2.3. Emotion Recognition (ER)

The PENN Emotion Recognition Task (ER40 [26]) was used to assess participants’ recognition of facial expressions. This is a computer-based task in which 40 color photographs of 20 male and 20 female faces expressing four basic emotions (happiness, sadness, anger, or fear) and no expression (neutral) were presented in pseudo-random order, with eight photos of each emotion. Participants were asked to select the correct emotion from the five choices listed alongside each photo as quickly and as accurately as possible. For each photograph, the depicted expression, the participants’ emotion choice, and their speed of responding were recorded. The ER40 outcome variables were: accuracy (correct responses: CR; misattribution errors/false positives: FP), and response times (for CR and FP). The ER40 has been used with adolescent populations [27] and is a valid and reliable measure of face emotion recognition [28]. 

### 2.4. Statistical Analyses

Descriptive analyses were performed on the participants’ mental health measures (K10 and SPHERE12) and social cognition (ER40) data (see Table 1). As diagnostics showed the mental health data was skewed, Spearman’s Rho bivariate correlations were conducted to examine the associations among the measures of mental health (K10 total scores and SPHERE-12 total scores, PSYCH, SOMA, and depression scales) (see Table 1). Spearman’s Rho correlations were also used to examine associations between the above mental health measures and emotion recognition for each of the five ER40 expressions (see Table 2). Mann–Whitney U tests were conducted to determine if there were differences in emotion recognition in those with threshold on the K10 or SPHERE-12 (including PSYCH, SOMA syndromes, or depression cases). Analyses were conducted using SPSS v24 (IBM, Armonk, NY, USA).

## 3. Results

### 3.1. Mental Health

Scores on the K10 showed that at the group level psychological distress was low, although 12.5% of the adolescents were at some level of risk for a mental illness (i.e., scores ≥ 20). Similar levels of risk for a mental disorder were found with the SPHERE-12 PSYCH scale: 10% of the sample met criteria for psychological symptomology. The SPHERE-12 total score showed that 40% of the participants reported elevated levels of psychological and somatic symptoms (scores > 5). Scores on the SOMA scale and depression caseness of the SPHERE-12 indicated that 17.5% of adolescents exhibited likelihood of a fatigue syndrome or depression caseness, respectively. Spearman’s Rho correlations among the measures of mental health are shown in Table 1. Correlation coefficients averaged 0.60 (range 0.37–0.91), indicating low likelihood of multicollinearity among the measures, with the exception of the high correlation between the SPHERE-12 Total scores and SPHERE-12 SOMA scores (r = 0.91).

### 3.2. Mental Health and ER: Anger

**Figure 1:**
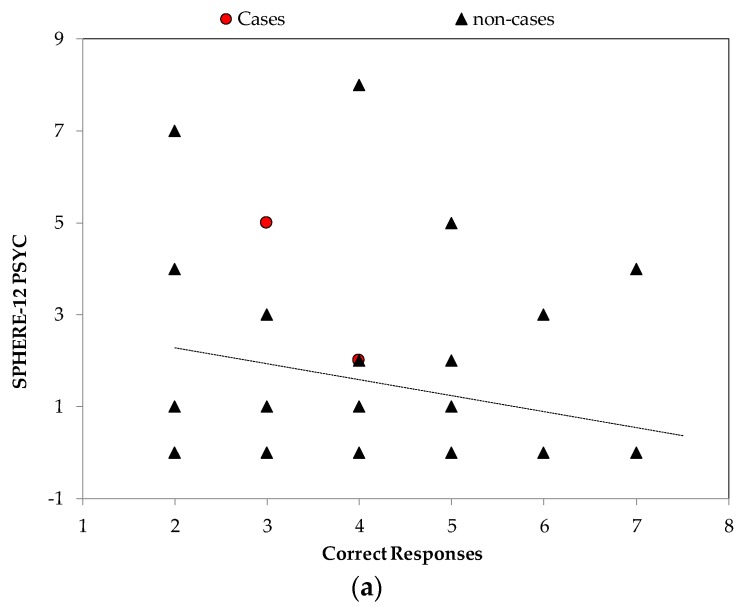

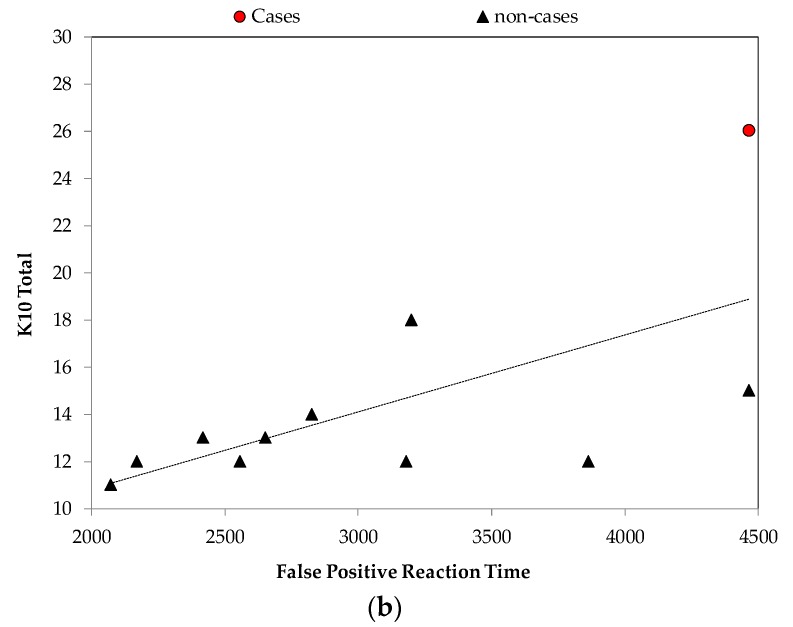
Angry Faces: scatter plots showing that (**a**) higher levels of psychological symptoms (SPHERE-12 PSYCH) are associated with fewer correct identifications of angry faces (r = −0.32) and that (**b**) higher levels of psychological distress (K10) are associated with slower reaction times for false positive for angry faces (r = 0.68 bottom panel).

Spearman’s Rho correlations between the mental health variables and emotion recognition are shown in Table 2. The correlations showed increased scores on the SPHERE-12 PSYCH scale were associated with fewer correct responses to angry faces (r = −0.32) compared to lower scores on the PSYCH scale (Figure 1, top panel). The correlations also revealed that the K10 scores were significantly associated with slower reactions times for false positive for anger affect (r = 0.68). That is, higher psychological distress scores predicted slower reaction times when mistaking other expressions as angry (Figure 1, bottom panel). There was also a significant negative association between the SPHERE-12 Total and false positives for anger (r = −0.28); whereby, increased symptomology predicted fewer mis-attributions of anger facial expressions (i.e., they were less likely to pick the other expressions as angry).

We next conducted a Mann–Whitney U Test to investigate whether youth who met criteria for likelihood of a mental disorder on the K10 (N = 5; 12.5%) or SPHERE-12 (N = 16; 40%) had differences in recognizing anger expressions, compared to those who did not. The results show no differences in accuracy of recognition of anger expressions, in participants who met criteria for likelihood of a mental disorder versus those who did not. However, the results indicated that SPHERE-12 depression cases (N = 7; 17.5%) showed significantly (*p* = 0.03) faster reaction times for identifying of angry faces (M = 1579.00 ms, SD = 248.79) compared to non-depressed youth (M = 2083.45 ms, SD = 819.84).

### 3.3. Mental Health and ER: Fear

For the K10, Spearman Rho’s correlations revealed that there was a negative significant association with reaction time for fear affect (r = −0.36); higher psychological distress was associated with faster identification of fear expressions. Similarly, there was a negative significant association between SPHERE-12 Total scores (r = −0.40) and SOMA scores (r = −0.41) and reaction time for fear; increased psychological and/or somatic symptoms were associated with faster reactions for fear facial expressions.

**Figure 2:**
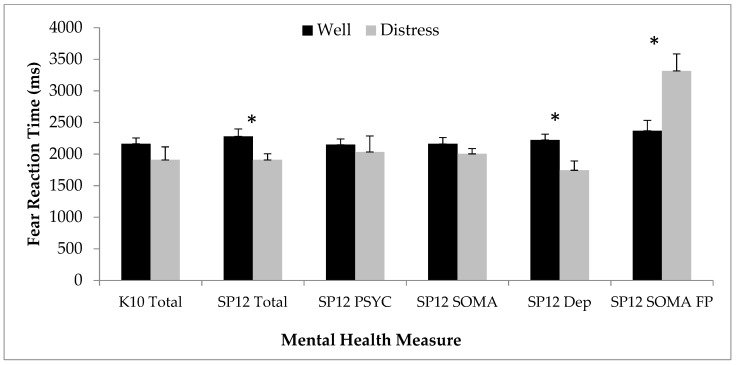
Fear Faces: bar graph showing differences in reaction times for recognition of fear affect or false positives for fear for psychologically well participants (black bars) vs. those with a possible mental disorder (grey bars). An asterisk above the bars denotes a significant difference (*p* < 0.05).

Mann–Whitney U Tests showed that youth whose SPHERE-12 Total scores indicated likelihood of a mental disorder had faster reaction times for fear expressions compared to non-distressed youth (*p* = 0.04; see Figure 2). With respect to the SPHERE-12 syndrome scales (PSYCH, SOMA, depression caseness; see Figure 2), Mann–Whitney U Tests indicated that there were no differences for the PSYCH syndrome scale between those who met threshold (N = 4; 10%) and those who did not. However, the SPHERE-12 Depression cases (N = 7; 17.5%) showed significantly faster reaction times for identification of fearful faces compared to non-depressed youth (*p* = 0.02) (Figure 2). Participants who met threshold for the SPHERE-12 SOMA syndrome (N = 7; 17.5%) had slower reactions time for false positives for fear faces compared to those who did not meet threshold. Mann–Whitney U Tests showed no differences in accuracy of fear recognition with respect to whether or not participants met criteria for likelihood of a mental disorder on the K10.

### 3.4. Mental Health and ER: Neutral

Spearman’s Rho correlations showed that for the SPHERE-12 Total (r = 0.35), SPHERE-12 PSYCH (r = 0.32), and depression cases (r = 0.40), there were positive correlations between correct responses for neutral faces and increased symptomology; higher scores predicted more accurate neutral face identifications. There was no association between K10 scores and processing of neutral faces.

Mann–Whitney U tests demonstrated significant differences in false positive neutral expression reaction times, with depression cases (N = 7; 17.5%) showing faster reaction times (M = 1858.21 ms, SD = 571.71) for misattribution of neutral expressions, compared with those who did not meet threshold (M = 2976.66 ms, SD = 331.47). There were no significant findings with the other SPHERE-12 scales or the K10 with respect to the neutral expressions.

### 3.5. Mental Health and ER: Happy and Sad

Spearman’s Rho correlations revealed that there were no significant associations between any of the mental health measures (K10, SPHERE-12 total, SPHERE-12 scales, or depression caseness) and any of the variables associated with recognition of happy faces; there were no processing differences regarding youth who met likelihood of a mental disorder vs. those who did not for happy expressions. Further, there were no significant associations between any of the mental health measures (K10, SPHERE-12 total, or SPHERE-12 scales, or depression caseness) and variables associated with sad face recognition; and there were no processing differences regarding youth who met likelihood of a mental disorder vs. those who did not for sad affect.

## 4. Discussion

The current study examined associations between mental health and facial emotion recognition in a community sample of 12-year-old adolescents. Our findings indicate that young people with higher levels of psychological distress (K10), psychological and/or somatic symptoms or depression caseness (SPHERE-12) exhibited a processing bias for negative facial affect. Higher psychological distress scores were associated with fewer correct responses to angry faces, suggesting an impairment in the ability to accurately identify this expression. However, higher scores on the SPHERE-12 and PSYCH scale were associated with fewer misattribution of other facial expressions as angry. Also, participants with poorer mental health or who met threshold for likelihood of a mental disorder, were faster at recognizing fearful expressions, made fewer misattributions of other expressions as fearful, and these misattributions were slower than participants with better mental health. The hypothesis that youth with elevated mental health problems would also have processing biases for sad affect, in line with other negative expressions, was not upheld. We also found that youths with increased psychological or somatic symptoms or depression caseness showed better processing of neutral faces, with more correct identifications compared to youth with lower levels of symptomology.

Importantly, these findings show that the processing bias for anger and fear expressions is associated with increased psychological symptomology in early adolescents (aged 12 years) without a mental illness diagnosis. These findings can be considered in relation to Keyes [29] perspective that, although related, mental health is more than the absence of a diagnosed mental illness. In his two continua model, mental health needs to be considered beyond pathological outcomes by exploring normal variation in emotional, psychological, and social wellbeing [30]. Wellbeing on the mental health continuum ranges from optimal functioning (flourishing) to poor functioning (languishing) [31]. Indeed, just as youth with mental illness show reduced daily functioning, so too do youth with languishing mental health [31]. This is the first study (we know of) with a community sample of 12-year-olds to demonstrate that youth exhibiting symptoms of poor mental health demonstrated a bias for negative emotion processing that are similar to those shown by youth with a mental disorder. Although the current study cannot explain the exact mechanism driving the emotion processing bias, this study demonstrates that the bias is not exclusive to individuals with a mental illness. Whether or not this negative-biased social-cognitive processing style may be an early indicator of later mental illness onset has yet to be investigated. The associations between fear recognition and the SPHERE PSYCH scale, but not on the general scales (K10 or SPHERE-12) or SPHERE SOMA scale, suggest that nature of the psychological distress may be specific to whether emotion processing problems arise.

Our finding with respect to poorer identification of angry faces in a community sample of young adolescents the SPHERE-12 PSYCH scale is consistent with the literature demonstrating that young people with mental illnesses exhibit poor processing of negative affect in other’s facial expressions [15]. We also showed that increased distress was related to faster processing (quicker reaction times) of fear expressions on the K10, SPHERE-12, and SOMA scale, suggesting hypervigilance for recognition of this expression. Likewise, participants with higher scores on the SPHERE-12 and PSYCH scale were also less likely to misattribute other facial expressions as angry, suggesting more accurate identification of negative affect. Although many studies demonstrate poorer processing of negative affect faces for young people with anxiety [15], this is not always the case. Our results are consistent with other studies, which have found enhanced processing of negative affect, particularly in youth with anxiety disorders [13] or depressive disorders [32]. These prior findings, combined with present findings, suggest that mental health may be associated with negative affect processing biases that may improve or reduce processing of fear expressions.

Our findings did not extend to a processing bias for sad affect found in prior research [16]. However, the current results are consistent with other research that also reported no impairments in processing sad faces in youth with anxiety [15] or bipolar disorder [33] who exhibited biases for fear or angry expressions. One explanation for this finding is that dissociable neural substrates may underlie responses to different expressions of negative effect. For example, Blair, Morris [34] showed activation of the left amygdala, and right inferior and middle temporal gyri to sad expressions in healthy participants. In contrast, the right orbitofrontal cortex was activated in response to angry expressions. For both sad and angry expressions the anterior cingulate cortex and right temporal pole were activated. In sum, this suggests differing, but inter-related, neural responses to these two negative expressions [34].

The current findings show that the processing bias for angry and fearful affect, as either impaired or enhanced, is evident in early adolescence (aged 12 years) exhibiting increased levels of psychological problems, even without mental illness diagnosis. It is possible that a negative-biased social-cognitive processing style may be an early indicator of later mental illness onset. The difficulty these young people had accurately recognizing angry facial expressions may contribute to the emergence and ongoing social challenges faced by individuals with mental illness. Interestingly, one study demonstrated that emotion recognition training in depressed individuals can reduce their negative affect processing bias, and that this has beneficial effects on mood [35]. However, although emotion recognition training was successful for people with anxiety, the reduced negative-face processing bias was not associated with reduced anxiety [36]. 

We also found that the young people with increased mental health problems showed better processing of neutral facial expressions which is consistent with another study showing enhanced processing of neutral expressions in youth with anxiety disorders compared to HC [15]. However, this finding contrasts with another study that found socially-anxious children were more likely to label neutral faces as exhibiting emotions [37]. It is possible that this discrepancy in findings could be due to the differing age of the participants across the studies, different diagnostic criteria, or the different assessment of facial emotion recognition utilized in each study.

Tracking the LABS cohort with regular and rigorous assessments of their mental health and wellbeing (including the Mini Neuropsychiatric Interview [38]) across adolescence, a developmental phase during which half of all adult mental illnesses emerge, will help determine whether the negative affect processing bias for anger and fearful expressions associated with psychological distress we found here becomes associated with specific mental disorder. These relationships are indicted by the associations between the nuanced psychological distress measure and scales used here and has been demonstrated in the broader literature with clinical populations.

LABS also has the capacity to explore the neurobiology that underpins psychological distress and emotion recognition in this young sample. We have previously shown that higher psychological distress scores are associated with reductions in specific hippocampal subfields and amygdala nuclei involved in fear and emotional regulation in 12-year-olds [39]. This structural change is accompanied by a resting-state functional decoupling of these limbic regions and the frontal cortex, circuitry heavily implicated in emotional regulation and processing [40]. Whether these structural and functional findings represent potential biomarkers for the emergence of mental disorders is unclear. However, our tracking of the developmental trajectory of these neuro circuitries from age 12 years throughout adolescence, via LABS, will provide new insights into the neuronal foundations of emergence of mental illness and divergence from healthy development.

The data presented in this manuscript has limitations. Firstly, the sample size is small, therefore, statistically significant findings should be interpreted with caution. However, as this data forms part of a larger longitudinal study, this paper highlights some key, early findings from data in the first year of the study. Second, there may have been a self-selection bias evident with youth who may have concerns regarding their mental health volunteering to participate in the study; although the rate of likelihood for a mental disorder in the current sample (12.5%) is consistent with population estimates in young people [21].

## 5. Conclusions

In summary, these results demonstrate associations between increased mental health problems and a processing bias for anger and fear negative facial expressions in a community sample of 12-year-olds. This supports the two-continua model of mental health and mental illness [31] and demonstrates that in a sample of youth without a clinical diagnosis, increased psychological problems may negatively impact on social cognition and functioning. This may be an early indicator of more serious mental health illnesses later in adolescence. As we continue to recruit young people into LABS and track them across adolescence, we will report on associations between youth with clinically diagnosed mental health problems and facial emotion recognition. We anticipate that data from LABS will inform the development of evidence-based youth mental health programs to support young people and their families.

## Figures and Tables

**Table 1:** Mental health measures: correlations, means, and likelihood of a mental disorder

Mental Health	K10	S 12 Total	SP12 PSYCH	SP12 SOMA	Mean (SD)	Possible Mental Disorder N (%)
K10					15.13 (4.45)	5 (12.5)
SP12 Total	0.61 **				4.08 (3.75)	16 (40)
SP12 PSYC	0.51 **	0.79 **			1.43 (2.02)	4 (10)
SP12 SOMA	0.57 **	0.91 **	0.52 **		2.65 (2.51)	7 (17.5)
SP12 Depression	0.37 *	0.65 **	0.65 **	0.46 **	0.18 (0.38)	7 (17.5)

* *p* < 0.05; ** *p* < 0.01; Possible Mental Disorder: K10 scores 21≥; Total score 6≥; PSYC score 2≥; SOMA score 3≥; depression caseness (PSYCH ≥ 2 and SOMA ≥ 3); SP12 = SPHERE-12.

**Table 2:** Correlations between mental health and emotion recognition

Facial Expressions					
Mental Health	Anger	Fear	Happy	Neutral	Sad
*Correct Responses (CR)*					
K10	−0.07	0.12	−0.08	0.10	−0.16
SP12 Total	−0.15	0.01	0.06	0.35 *	−0.17
SP12 PSYC	−0.32 *	0.03	−0.02	0.32 *	−0.21
SP12 SOMA	−0.06	−0.03	0.07	0.26	−0.18
SP12 Depression	−0.16	−0.06	0.15	0.40 *	−0.04
*False Positives (FP)*					
K10	−0.11	−0.12	−0.10	0.11	−0.10
SP12 Total	−0.28 *	0.10	−0.22	0.07	−0.06
SP12 PSYC	−0.42 **	0.23	−0.11	0.17	−0.07
SP12 SOMA	−0.17	−0.05	−0.18	0.18	−0.09
SP12 Depression	−0.15	0.15	−0.09	−0.01	0.01
*Reaction Time for CR*					
K10	−0.07	−0.36 *	0.001	0.29	0.13
SP12 Total	−0.003	−0.40 *	0.02	0.13	−0.06
SP12 PSYC	−0.02	−0.31	0.01	0.04	−0.13
SP12 SOMA	−0.20	−0.41 **	0.06	0.16	−0.04
SP12 Depression	−0.01	−0.12	0.01	0.05	−0.21
*Reaction Time for FP*					
K10	0.68 *	−0.10	−0.12	−0.03	0.02
SP12 Total	0.50	−0.02	−0.04	−0.24	0.10
SP12 PSYC	0.54	−0.18	−0.09	−0.23	0.01
SP12 SOMA	0.50	0.11	0.07	−0.22	0.11
SP12 Depression	0.50	−0.22	−0.10	−0.16	−0.05

* *p* < 0.05; ** *p* < 0.01, CR = correct responses; FP = false positives.
